# The reduction of XIAP is associated with inflammasome activation in RPE: implications for AMD pathogenesis

**DOI:** 10.1186/s12974-019-1558-5

**Published:** 2019-08-22

**Authors:** Jiangyuan Gao, Jing Z. Cui, Aikun Wang, Hao Hang Rachel Chen, Alison Fong, Joanne A. Matsubara

**Affiliations:** Department of Ophthalmology and Visual Sciences, Faculty of Medicine, Eye Care Centre, University of British Columbia, 2550 Willow Street, Vancouver, BC V5Z 3N9 Canada

**Keywords:** Age-related macular degeneration, XIAP, Caspase-1, Inflammasome, Pyroptosis, And retinal pigment epithelium

## Abstract

**Background:**

Age-related macular degeneration (AMD) is a multifactorial chronic disease of the eye. Several candidate pathways have been hypothesized to play a role in AMD pathogenesis. Our work and those of others suggests inflammasome activity as a mechanism associated with retinal pigment epithelial (RPE) cell demise. X-linked inhibitor of apoptosis protein (XIAP), an anti-apoptosis factor, has recently been shown to regulate inflammasome activity in non-ocular cells. The purpose of this study is to characterize XIAP’s regulatory role in RPE.

**Methods:**

Protein lysates of eye tissues from rats (vinpocetine- or aurin tricarboxylic acid complex-treated, ATAC, vs naïve) and mice (wild type vs *Caspase-4*^*−/−*^) were utilized to analyze XIAP protein levels. Immunohistochemistry was used to detect NLRP3 levels in the RPE layer. In vitro inflammasome activation on RPE cells was achieved with L-leucyl-L-leucine methyl ester (Leu-Leu-OMe) stimulation. Levels of XIAP mRNA and 18S RNA were quantified by RT-PCR. Cell culture supernatants were tested directly for secreted IL-1β by ELISA or concentrated for the detection of secreted IL-18 by western blot. Protein lysates from RPE in cell culture were collected for the measurement of cleaved caspase-1 p20, XIAP, and GAPDH. Data are presented as Mean ± SD. *p* < 0.05 is considered statistically significant.

**Results:**

The XIAP protein level was significantly increased when the inflammasome was inhibited at the “activation” step by ATAC, but not the “priming” step, in vivo. Concomitantly, NLRP3 immunoreactivity was lower in the RPE layer of animals fed with ATAC. In mice where caspase-1 cleavage was impaired by the genetic deficiency in caspase-4, the XIAP protein level increased in eye tissues. In RPE cell culture, Leu-Leu-OMe stimulation led to caspase-1 cleavage, cytokine secretion, and XIAP reduction, which can be abolished by Z-YVAD-FMK. When XIAP siRNA was given as a pre-treatment to RPE in vitro, Leu-Leu-OMe induced IL-1β/IL-18 secretion was enhanced, whereas overexpressing XIAP reduced IL-1β secretion under inflammasome activation, both compared to controls cells.

**Conclusions:**

Together, these data suggest XIAP-mediated inhibition of inflammasome activity in RPE may provide insights into the biological consequences of inflammasome activation in RPE and reveals the caspase-1/XIAP/IL-1β/IL-18 axis as a target for broader applications in AMD biology and treatment design.

**Electronic supplementary material:**

The online version of this article (10.1186/s12974-019-1558-5) contains supplementary material, which is available to authorized users.

## Background

Age-related macular degeneration (AMD) is a complex disease with various risk factors contributing to its pathogenesis. Despite the fact that the exact molecular basis underlying AMD is not yet fully understood, several candidate cellular and biochemical pathways associated with its development have been hypothesized. As a defense mechanism of the innate immune system, an intracellular, multi-protein complex, known as the inflammasome, possesses versatile activation capacities, and subsequent pro-inflammatory action. The inflammasome complex is not limited to immune cells; in fact, being the first tissues responding to injuries and pathogens, epithelial tissues have been shown to contain inflammasomes [1]. The most widely studied inflammasome is the Nod-like receptor protein 3 (NLRP3) inflammasome, which responds to a diversity of danger-associated molecular patterns (DAMPs), including bacteria, pore-forming toxins, uric acid crystals, particulate aggregates, and adenosine triphosphate. When activated, precursor caspase-1 (pro-caspase-1) cleaves itself into active fragments to facilitate the secretion of cytokines IL­1β and IL­18. In addition, the NLRP3 inflammasome activation can also trigger proteolytic processing of gasdermin D (GSDMD), a pore-forming protein that executes pyroptosis [2]. As to other inflammasome subtypes (e.g., NLRP1, NLRC4, AIM2), despite the difference in molecular composition, caspase-1 cleavage is also required for their activation [3].

Using functional data of caspase-1 cleavage and cytokine secretion, our earlier work and those of others, point towards the activation of inflammasome as a pathway associated with retinal pigment epithelium (RPE) cell demise [4–7]. Since the RPE cell is susceptible to molecular and cellular dysregulation during the aging process, just like other long-lived postmitotic cells [8, 9], it is critical to precisely control the level of inflammasome activity in order to avoid adverse effects. Clinically, inflammasome antagonists are being explored as novel therapeutics for treating human immune diseases [10, 11]. The inhibition of inflammasome activity can potentially be achieved at four different levels along its activation pathway [10, 12]. These include blocking cell membrane receptors (e.g., P2X7 receptor for ATP), controlling cytoplasmic second messengers (e.g., K^+^, cathepsin B, reactive oxygen species), preventing inflammasome components from assembling, and antagonizing released cytokine products and/or their cognate receptors. Given the fact that inflammasomes respond to a variety of activation signals, it is arguably more beneficial to slow down or stop the inflammasome assembly and subsequently, the release of pro-inflammatory cytokines, rather than suppress the multitude of potential activation signals.

The X-linked inhibitor of apoptosis protein (XIAP) is a classic and potent anti-apoptosis factor, previously shown to be involved in inflammasome activation in non-ocular cells [13–15]. Macrophages from *Xiap*^*−/−*^ mice exhibit enhanced caspase-1 cleavage and IL-1β secretion when stimulated for inflammasome activation, compared to the wild type controls [13]. *Nlrp3*^*−/−*^ dendritic cells present elevated XIAP expression, suggesting a potential negative regulation between the NLRP3 inflammasome and XIAP [14]. In rat spinal cord neurons, XIAP constitutively binds to the inactive inflammasome complex. Upon spinal cord injury, the inflammasome is activated and XIAP is degraded [15]. More intriguingly, our recent finding demonstrated a decline in XIAP protein level in rodent eyes receiving intravitreal injections of an inflammasome-activating molecule, amyloid beta (Aβ) [5]. Hence, this study aims to further understand XIAP’s role in inflammasome regulation in RPE cells.

## Methods

### Animal tissue samples

All animal procedures were approved by the Animal Care Committee of the University of British Columbia and conformed to the guidelines of the Canadian Council on Animal Care and in accordance with the Resolution on the Use of Animals in Research of the Association of Research in Vision and Ophthalmology. RPE/choroid protein lysates were used in this study to evaluate the association between XIAP and the inflammasome [16, 17]. These protein samples include Aβ_1–40_ + vinpocetine (vinpo) vs Aβ_1–40_ + vehicle (*N* = 5 per group, see Liu et al. for details [16]), and aurin tricarboxylic acid complex (ATAC) vs Control (*N* = 6 per group, see Zhao et al. for details [17]). In addition, wild type (C57BL/6J, Jackson Laboratory) and *Caspase-4*^*−/−*^ mice (B6.129S4(D2)-*Casp4*^*tm1Yuan*^/J, Jackson Laboratory) were also sacrificed for whole eye protein lysates (*N* = 4 per group).

### Isolation and culture of primary human fetal RPE cells

Primary RPE cells were isolated from human fetal donor eyes following previously published protocols [18–20]. All procedures were approved by and performed under the guidelines of the Clinical Research Ethics Board at the University of British Columbia. The fetal donor eye tissues consented for research had no known pathology and were used in compliance with the Declaration of Helsinki. In the following experiments, passage 4–6 primary RPE cells were used.

### NLRP3 immunohistochemistry

Rat eye sections (Control vs ATAC fed) from both 7.5 months old and 11.5 months old groups were deparaffinized for proteinase K mediated antigen retrieval. Sections were then blocked with 0.3% hydrogen peroxide and 5% normal goat serum before overnight incubation with Cryo-2 anti-NLRP3 antibody (Adipogen, Switzerland, Table 1). Replacement of the primary antibody with mouse IgG2b isotype antibody was used as negative control. Next, the biotinylated goat anti-mouse secondary antibody was used to visualize positive NLRP3 immunoreactivity in the presence of 3-amino-9-ethylcarbazole substrate (Vector Labs, Burlingame, CA). A 0–3-point grading scheme was followed as previously described [5]. The immunoreactivity scores were averaged and compared between the two treatment groups at each time point.

**Table 1:** List of primary antibodies used

Antigen	Antibody (clone no.)	Dilution	Source
NLRP3	Mouse monoclonal (Cryo-2)	1:200	Adipogen, Switzerland
Interleukin-18 (IL-18)	Rabbit polyclonal (H-173)	1:1000	Santa Cruz Biotechnology, Dallas, TX
X-linked inhibitor of apoptosis (XIAP)	Mouse monoclonal (48/hILP/XIAP)	1:1000	BD Transduction Laboratories, San Jose, CA
Caspase-1	Mouse monoclonal (661228)	1:1000	R&D Systems, Minneapolis, MN
GAPDH	Mouse monoclonal (6C5)	1:10,000	EMD Millipore, Billerica, MA

### In vitro model of inflammasome activation

To fully assess the molecular mechanisms underlying the inflammasome activation in RPE, low-passage ARPE-19 cells and primary human fetal RPE cells were used. Cells were maintained in complete culture medium, including DMEM/F12, high glucose, 1% penicillin/streptomycin and 10% fetal bovine serum (FBS), and incubated at 37 °C in a humidified atmosphere of 95% air and 5% CO_2_. To activate the inflammasome, RPE cells were seeded in complete culture medium in 6-well plates at a density of 6 × 10^5^ cells per well and cultured for overnight. Then the cells were washed twice in DMEM/F12 medium before subject to various stimulation conditions. L-leucyl-L-leucine methyl ester (Leu-Leu-OMe; Chem-Impex International, Wood Dale, IL), a lysosomotropic agent endocytosed by cells and converted into (LeuLeu)_n_-OMe (*n* > 3) in lysosomes causing lysosomal destabilization [21], was applied with or without the pre-incubation of recombinant human IL-1α at 10 ng/mL (“priming”). Leu-Leu-OMe concentration of 1 mM was proven effective to destabilize RPE lysosomes according to previously established protocol [6]. After 3 h of Leu-Leu-OMe stimulation (1 mL/well at 1 mM), cell culture supernatants were collected and cells were lysed in 200 μL/well ice-cold RIPA buffer supplemented with protease inhibitors. To prevent inflammasome activation, a cell-permeable, irreversible caspase-1 inhibitor, Z-YVAD-FMK (R&D systems, Oakville, ON) was added onto the cell cultures at either 1-μM or 20-μM concentration 1 h before Leu-Leu-OMe stimulation. After centrifugation at 14,800 rpm, 4 °C for 20 min, 900 μL of cell-free culture supernatant and 200 μL of cell lysates for each stimulation conditions were stored at − 20 °C.

### XIAP siRNA knockdown

The validated *Silencer*® Select human XIAP 21-mer siRNA, which have been functionally tested to reduce human XIAP gene expression, and the recommended *Silencer*® Select negative control siRNA (Ng siRNA) that is experimentally tested not to target any gene product were purchased from Life Technologies. Preliminary tests were run in-house to assure the minimal off-target effects and the efficiency of siRNA delivery. ARPE-19 cells transfected with or without the negative control siRNA did not exhibit significant difference in XIAP mRNA levels (Additional file 1: Figure S1). The XIAP siRNA knockdown procedures were conducted as per the manufacturer’s protocols. Briefly, both the ARPE-19 cells and the primary RPE cells in complete culture medium were seeded into 6-well plates at a density of 6 × 10^5^ cells per well 1 day before the siRNA transfection. At the time of transfection, RPE cells were at ~ 80% confluence. The cells were first washed twice in DMEM/F12 only medium and then 3 mL of serum-/antibiotics-free DMEM/F12 medium was added onto each well. A lipid-based transfection reagent, RNAiMAX (Invitrogen), was used to prepare the siRNA-lipid complex in serum-/antibiotics-free DMEM/F12 medium. A series of three different XIAP siRNA final concentrations were tested during a 48-h incubation period: 2.5 nM, 5.0 nM, and 10 nM, of which the lowest effective dose (2.5 nM) was determined by reverse transcription PCR (RT-PCR) of XIAP mRNA in ARPE-19 cells (Fig. 6a). Similarly, the 2.5-nM concentration of XIAP siRNA was also proven effective in primary RPE cells over an incubation period as short as 24 h (Additional file 2: Figure S2). Therefore, for the rest of the in vitro XIAP siRNA experiments, a final concentration of 2.5 nM was used throughout. The effectiveness of siRNA knockdown was further assured by XIAP western blot.

### XIAP plasmid construct and overexpression in RPE cells

The XIAP plasmid was modified from its original version that was a gift from Dr. Catherine Tsilfidis (University of Ottawa) in order to be better expressed in RPE cells: the generic mammalian cytomegalovirus (CMV) promoter was engineered to drive the transcription of hemagglutinin-XIAP fragment (HA-XIAP) in an eukaryotic expression vector pcDNA3.1(+) (Invitrogen, Carlsbad, CA). Restriction enzyme sites were introduced to flank the HA-XIAP fragment (BamHI on the 5′ end, XhoI on the 3′ end) using the following PCR primer pair (5′➔3′). BamHI-HA-XIAP: TTTCGGATCCATGTACCCATAC; HA-XIAP-XhoI: TCTCCTCGAGGGGCTTAAGATCTATTTAAGAC. To construct the expression vector pcDNA3.1(+)/HA-XIAP, both HA-XIAP and the vector pcDNA3.1(+) insert DNAs were digested by restriction enzymes BamHI and XhoI, and then separated by 1% agarose gel electrophoresis. The target bands were incised for DNA purification (QIAquick PCR Purification Kit, Qiagen, Toronto ON, Canada) and ligation. The pcDNA3.1(+)-HA-XIAP ligator was then transformed into DH5α *E. coli* competent cells and cultured on an ampicillin-containing LB-agar plate for overnight. Only single, large ampicillin-resistant *E. coli* colonies were inoculated into ampicillin-containing LB medium for plasmid amplification and extraction (QIAamp DNA Mini Kit, Qiagen, Toronto, ON, Canada). To assure the accuracy of XIAP sequence, the extracted plasmid was further analyzed by sequencing (primer sequences: 5′-GGCTAACTAGAGAACCC-3′ and 5′-CCTGGTCAGAACACAG-3′).

To overexpress XIAP in primary RPE cells, the XIAP plasmid (pcDNA3.1(+)-HA-XIAP) and its vector control (pcDNA3.1(+) plasmid without the insertion of the HA-XIAP transgene) were transfected into the cells following our established protocol [16]. Briefly, transfection was performed in the 6-well plate format, using 2 μg total DNA for each well. A 24-h transfection incubation regime was applied, followed by 24-h IL-1α priming (10 ng/mL) and 3-h inflammasome activation by Leu-Leu-OMe (1 mM).

### Reverse transcription PCR (RT-PCR)

Total RNA of ARPE-19 cells and primary RPE cells from different stimulation condition groups (in triplicates) were extracted using ultRNA Column Purification kit (Applied Biological Materials). One microgram total RNA from each well was reverse transcribed into cDNA using the High-Capacity cDNA Reverse Transcription kit (Applied Biosystems). RT-PCR was carried out on the 7500 Fast Real-time PCR System (Applied Biosystems) using the SYBR Green Master Mix (Applied Biosystems) and followed the cycling conditions: 95 °C for 30 s, 50 °C for 30 s, 72 °C for 30 s, 40 cycles. RT-PCR primer sequences can be found in Table 2. Melting curve analysis was automatically performed right after the cycles’ completion. The results were expressed as mRNA fold-change relative to the control group after normalization to the reference gene, using the 2^−ΔΔCT^ method.

**Table 2:** List of RT-PCR primer sequences used for ARPE-19 cells

Human Gene	Forward Primer (5′-3′)	Reverse Primer (5′-3′)
X-linked inhibitor of apoptosis (XIAP)	CAGACTATGCTCACCTAACC	CCAAAAGTAAAGATCCGTGC
18S RNA (reference gene for siRNA assays)	GTAACCCGTTGAACCCCA	CCATCCAATCGGTAGTAG

### IL-1β immunoassay

To measure the level of secreted IL-1β in the culture medium, primary RPE cells were primed by recombinant human IL-1α at 10 ng/mL for 24 h before the start of various stimulation conditions. The resulting cell culture medium supernatants were collected and centrifuged at 148,000 rpm for 20 min to remove cellular debris. Thirty microliters of each cell-free supernatant sample was mixed with equal volume of sample diluent and loaded onto a pre-wetted V-PLEX human IL-1β immunoassay plate (Meso Scale Diagnostics, Rockville, MD) for overnight incubation at 4 °C on a microplate shaker at the speed of 700 rpm. A SULFO-TAG anti-human IL-1β antibody was used to capture the secreted IL-1β for detection. The samples’ IL-1β concentrations were calculated based on the standard curve and accounted for the dilution factor. The human IL-1β immunoassay used here is a highly sensitive immunoassay that outperforms other detection methods, such as fluorescence or absorbance used in traditional ELISA, due to its very low backgrounds, high sensitivity, and broad dynamic range which allowed for the detection of IL-1β with lower limits of detection in the < pg/mL (0.008 pg/mL) range.

### Western blot

To detect the secreted IL-18 and caspase-1 in ARPE-19 cell culture supernatant after Leu-Leu-OMe stimulation, 10 μL of the StrataClean resin solution (hydroxylated silica particles, Agilent Technologies, Santa Clara, CA) was added into each of the 900-μL culture supernatant samples. Next, the sample-resin slurry was vortexed to achieve a homogeneous distribution and incubated them at 4 °C for 2 h on a 360° rotator. Concentrated supernatant proteins were then collected by pelleting the StrataClean resin at 14,800 rpm, 4 °C for 20 min and the non-protein liquid phase removed. The resin-protein complexes were then resuspended in 1x reducing loading buffer (40 μL per sample) and denatured at 95 °C for 5 min to release the bound proteins. The resin-sample-loading buffer solution was pelleted, and the upper phase protein-containing solution was loaded onto gels.

To detect the intracellular proteins, cell protein lysates were quantified by BCA assay (Pierce, Thermo Fisher Scientific) for total protein concentrations and run on gels under reducing conditions. Established blotting procedures were followed to visualize the proteins of interest [17]. A list of primary antibodies used in western blot is included (Table 2). As an internal protein loading control, GAPDH was detected. The protein band intensity of XIAP (57 kD) and GAPDH (36 kD) was individually measured using Image J (NIH, Bethesda, MD) and converted into ratios relative to GAPDH. The final relative intensity of XIAP was normalized to the control group.

### Statistical analysis

Data are presented as Mean ± SD. All experiments were repeated three times. Statistical analysis was performed using GraphPad Prism version 7 (GraphPad Software). To compare two groups, Mann-Whitney *U* test was used for western blot and immunohistochemistry analysis of animal tissue samples. To compare more than two groups, a Kruskal-Wallis and a post hoc Dunn’s multiple comparison test was used for western blot analysis of ARPE-19 cell lysates, whereas a one-way ANOVA and a post hoc Bonferroni multiple comparison test was used for RT-PCR analysis of XIAP mRNA levels after siRNA knockdown in both ARPE-19 and primary RPE cells, and also for the IL-1β ELISA assay in primary RPE cells. Statistical significance level was set at *p* < 0.05.

## Results

### XIAP is involved in the inflammasome activation step

**Fig. 1:**
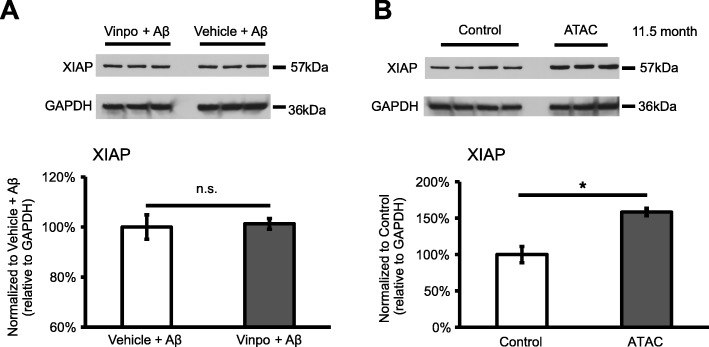
**a**, **b** XIAP protein level is associated with inflammasome activation step. XIAP western blot was performed using RPE/choroid protein lysates from previous studies [16, 17]. Significant difference in XIAP protein levels was observed between ATAC-treated animals and non-treated controls (*N* = 6, Mann-Whitney, *p < 0.05). There was no difference in XIAP levels with administration of vinpocetine (vinpo, a NF-κB blocker) compared to control

**Fig. 2:**
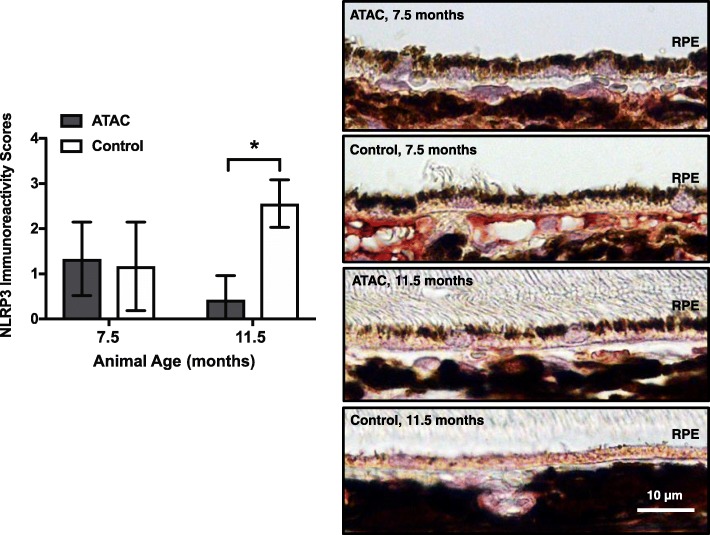
ATAC reduces NLRP3 in RPE. ATAC-fed rats exhibited a significantly lower level of NLRP3 immunoreactivity (red, AEC substrate) in RPE cells compared to the control group at 11.5 months (Mann-Whitney, *p < 0.05). No significant difference of NLRP3 immunoreactivity was observed between the ATAC-fed and control animals at the younger age (7.5 months). Representative micrographs were taken under × 40 objective lens. Scale bar, 10 μm

**Fig. 3:**
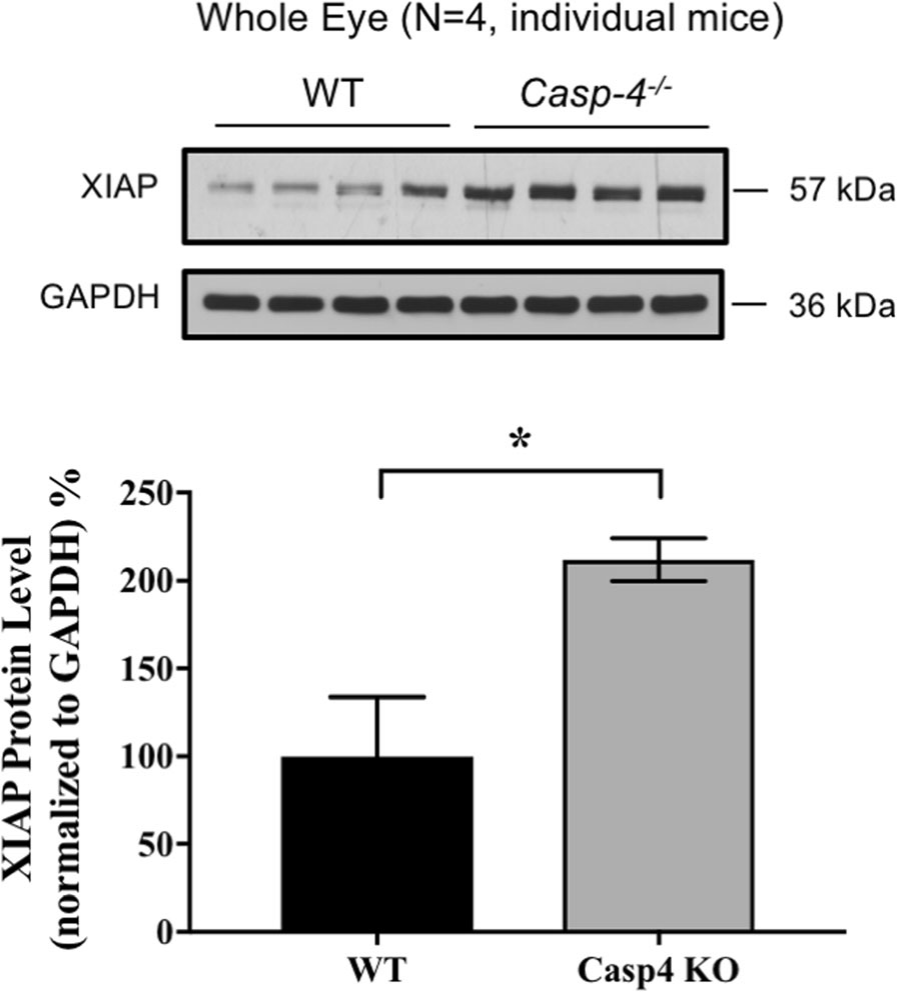
XIAP level increases in the eyes of *Caspase-4* knockout mice that have impaired processing of Caspase-1 precursor protein. Deficiency in Caspase-1 cleavage leads to a significant increase (~ twofold) in the XIAP protein level in the whole eye protein lysates from the *Caspase-4* knockout mice (*Casp-4*^*−/−*^) compared to the wildtype controls (WT) (Mann-Whitney, *p < 0.05)

The concept that XIAP may regulate the inflammasome pathway is novel. There is little knowledge as to what role XIAP might play in the immune regulation, especially in the inflammasome cascade. In our previous study, we established an in vivo model of inflammasome activation in RPE/choroid, demonstrating robust caspase-1 immunoreactivity and cytokine secretion (IL-18 and IL-1β) [5]. Intriguingly, in that model, we also found evidence of concomitant XIAP reduction at both the mRNA and protein levels when the inflammasome was activated. This prompted us to further investigate XIAP and its potential association with inflammasome activity. Using vinpocetine, a potent NF-κB blocker, we were able to show a strong inhibition of caspase-1 cleavage [16]. However, when we tested the same set of protein samples for XIAP by western blotting, there was no significant difference in XIAP protein abundance, suggesting that XIAP is not affected by the NF-κB “priming” signal (Fig. 1a). We then looked at the involvement of XIAP in the pathway associated with the “activation” signal. From our earlier study, membrane attack complex (MAC) induced caspase-1 cleavage by the “activation” pathway, and this was prevented by ATAC, a drug known to disrupt MAC formation [17]. By testing these samples, we found that XIAP protein level was increased by more than 50% and NLRP3 immunoreactivity was markedly reduced when animals were given ATAC, compared to controls at 11.5 months (Figs. 1b and 2). Furthermore, in *Caspase-4*^*−/−*^ mice that do not have functional cleaved caspase-1, XIAP protein levels were twofold higher compared to wild type mice (Fig. 3). Together, these data indicate that XIAP is regulated by the inflammasome activity.

### Reduction of XIAP protein under inflammasome activation in vitro

**Fig. 4:**
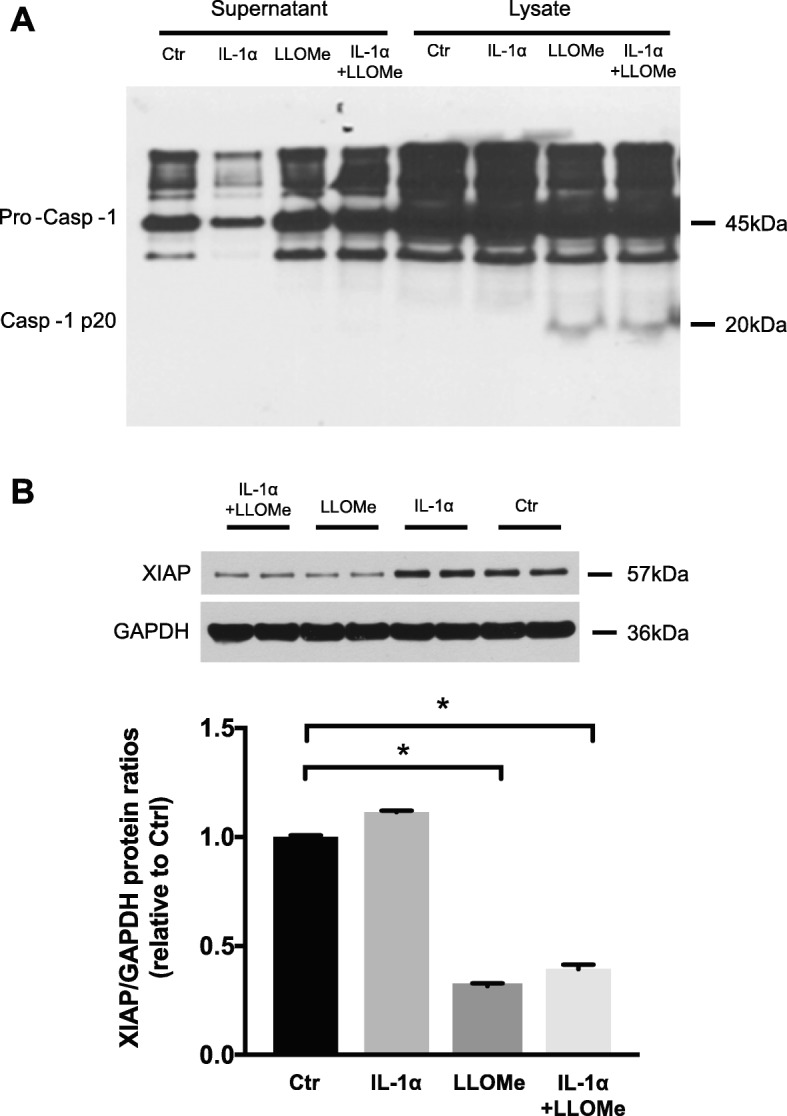
Leu-Leu-OMe stimulation alone is sufficient to activate inflammasome and reduce the XIAP protein level. **a** Caspase-1 western blot was performed using ARPE-19 cell culture supernatants and cell lysates from four stimulation groups: non-stimulated RPE, IL-1α primed RPE, Leu-Leu-OMe (LLOMe) stimulated RPE, IL-1α primed, and LLOMe stimulated RPE. The cleaved caspase-1 (Casp-1) p20 bands were observed under LLOMe stimulation regardless of IL-1α priming. **b** XIAP protein levels were studied using cell lysates from the same groups, where LLOMe stimulation was able to decrease XIAP in RPE cells with or without IL-1α priming, compared to non-stimulated RPE cells or cells only primed with IL-1α (*N* = 3, Kruskal-Wallis, *p < 0.05)

**Fig. 5:**
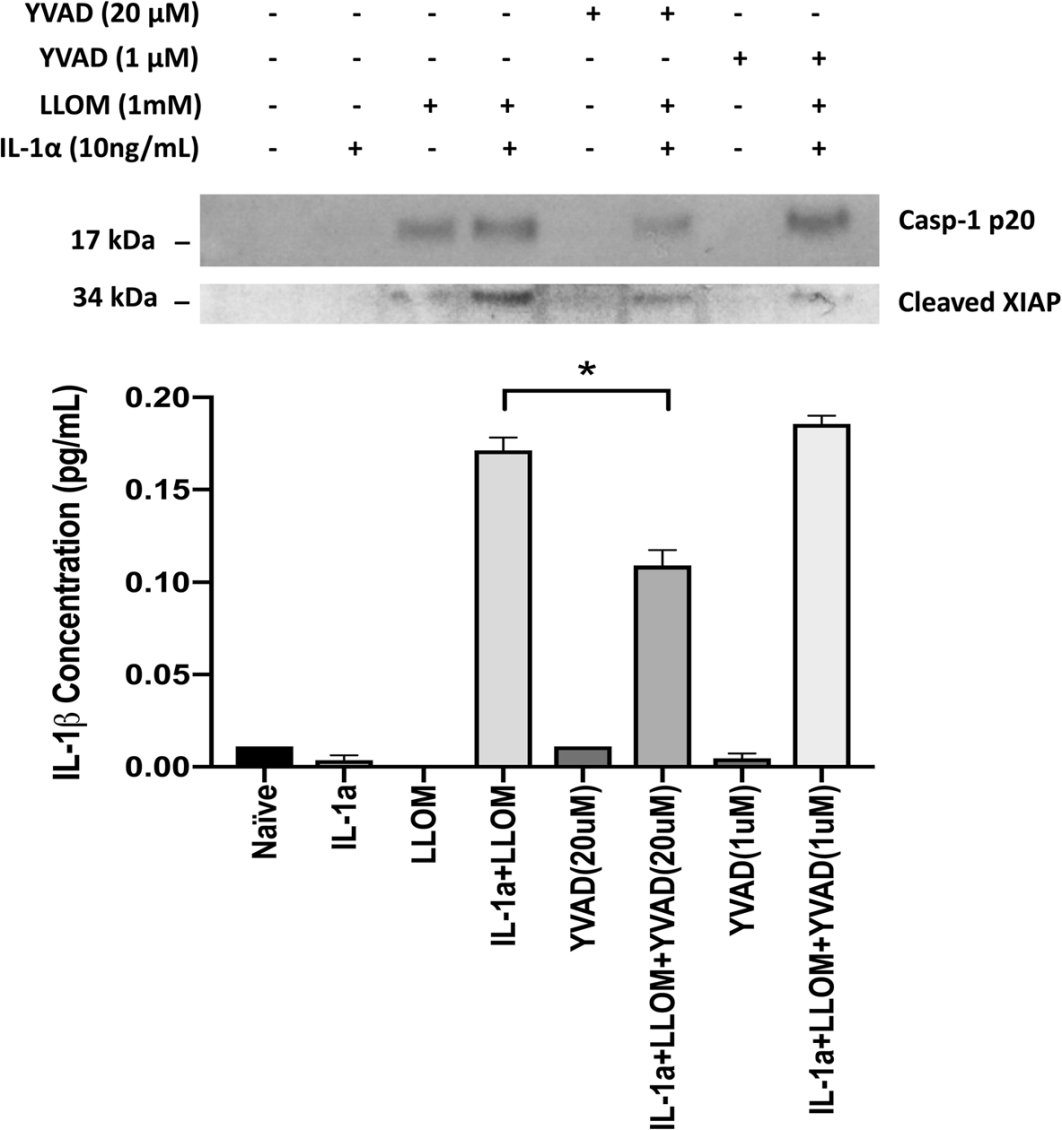
YVAD inhibits Caspase-1 and XIAP cleavage and lowers IL-1β secretion in RPE. Caspase-1 p20 bands were found under all LLOMe-containing stimulation groups, the intensity of which was reduced by 1 h pre-incubation of YVAD at 20 μM but not 1 μM. Similarly, IL-1β’s secretion was also affected by YVAD inhibition, where 20 μM YVAD reduced more than 30% of secreted IL-1β in primary RPE cell culture supernatants (One-way ANOVA, *p < 0.05). When inflammasome was active, evident by the presence of Caspase-1 p20 band and secreted IL-1β, XIAP was cleaved into a small fragment of ~ 34 kDa in molecular weight. The change of XIAP cleavage level followed that in Caspase-1 p20 and IL-1β

To verify this inverse correlation between XIAP and the inflammasome activity, we tested ARPE-19 cells under four conditions: non-stimulated ARPE-19 cells (Ctrl); ARPE-19 cells primed with IL-1α for 48 h; ARPE-19 cells stimulated with Leu-Leu-OMe for 3 h; ARPE-19 cells first primed with IL-1α for 48 h and subsequently stimulated with Leu-Leu-OMe for 3 h. Comparisons among the four groups revealed strong cleaved caspase-1 p20 bands in the cell lysates from Leu-Leu-OMe alone and IL-1α + Leu-Leu-OMe combined stimulation groups, confirming inflammasome activity. Comparing XIAP protein levels in these four groups revealed that there was a greater than 50% decrease in XIAP band intensity in the presence of Leu-Leu-OMe stimulation, compared to Ctrl and IL-1α priming alone groups (*p < 0.05). However, IL-1α + Leu-Leu-OMe combined stimulation did not further reduce the XIAP level (Fig. 4). To further test the hypothesis that XIAP’s reduction is a result of inflammasome activation, we applied a cell-permeable, irreversible caspase-1 inhibitor, Z-YVAD-FMK. We found that 20 μM of Z-YVAD-FMK, when combined with IL-1α + Leu-Leu-OMe stimulation, was able to lower Caspase-1 p20 cleavage, which in turn reduced the generation of cleaved XIAP (34 KDa) and IL-1β secretion, compared to the IL-1α + Leu-Leu-OMe group alone. When Z-YVAD-FMK was used at a lower concentration (1 μM), both Caspase-1 p20 cleavage and IL-1β secretion were similar to their levels in the IL-1α + Leu-Leu-OMe group, suggesting the effective blockage of caspase-1 p20 cleavage helps preserve XIAP (Fig. 5).

### XIAP cleavage affects IL-18 and IL-1β release under inflammasome activation

**Fig. 6:**
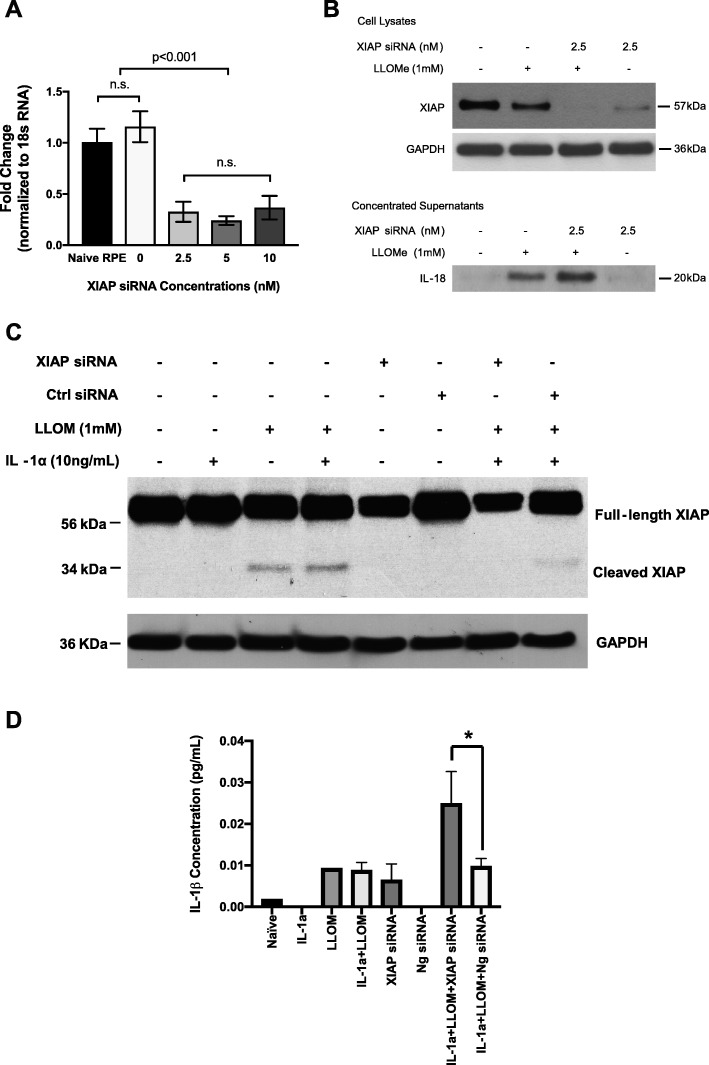
XIAP siRNA knockdown in RPE cells enhances both IL-18 and IL-1β release. **a** The selection of effective XIAP siRNA concentration was determined by RT-PCR assays in ARPE-19 cells, where a lower dose of XIAP siRNA (2.5 nM) achieved more than 75% inhibition on XIAP mRNA. Increasing the XIAP siRNA concentrations (5 nM or 10 nM) did not provide additional inhibition (*N* = 3, one-way ANOVA, *p* < 0.001). **b** Cell lysates and culture medium supernatants from non-primed ARPE-19 cells under different stimulation conditions were collected for protein analysis. Leu-Leu-OMe (LLOMe) stimulation alone triggered IL-18 secretion and XIAP reduction. Cells pre-treated with XIAP siRNA, then followed by LLOMe stimulation, exhibited more IL-18 and further decrease of XIAP, compared to LLOMe alone group. **c** XIAP cleavage (from a full-length 57 kDa protein to a ~ 34 kDa fragment) was present under LLOMe induced inflammasome activation but was abolished by XIAP siRNA pre-incubation. **d** When stimulated with a combination of IL-1α and LLOMe, a higher secreted level of IL-1β was present in the XIAP siRNA-treated primary RPE cells, compared to the control siRNA group (One-way ANOVA, *p < 0.05)

**Fig. 7:**
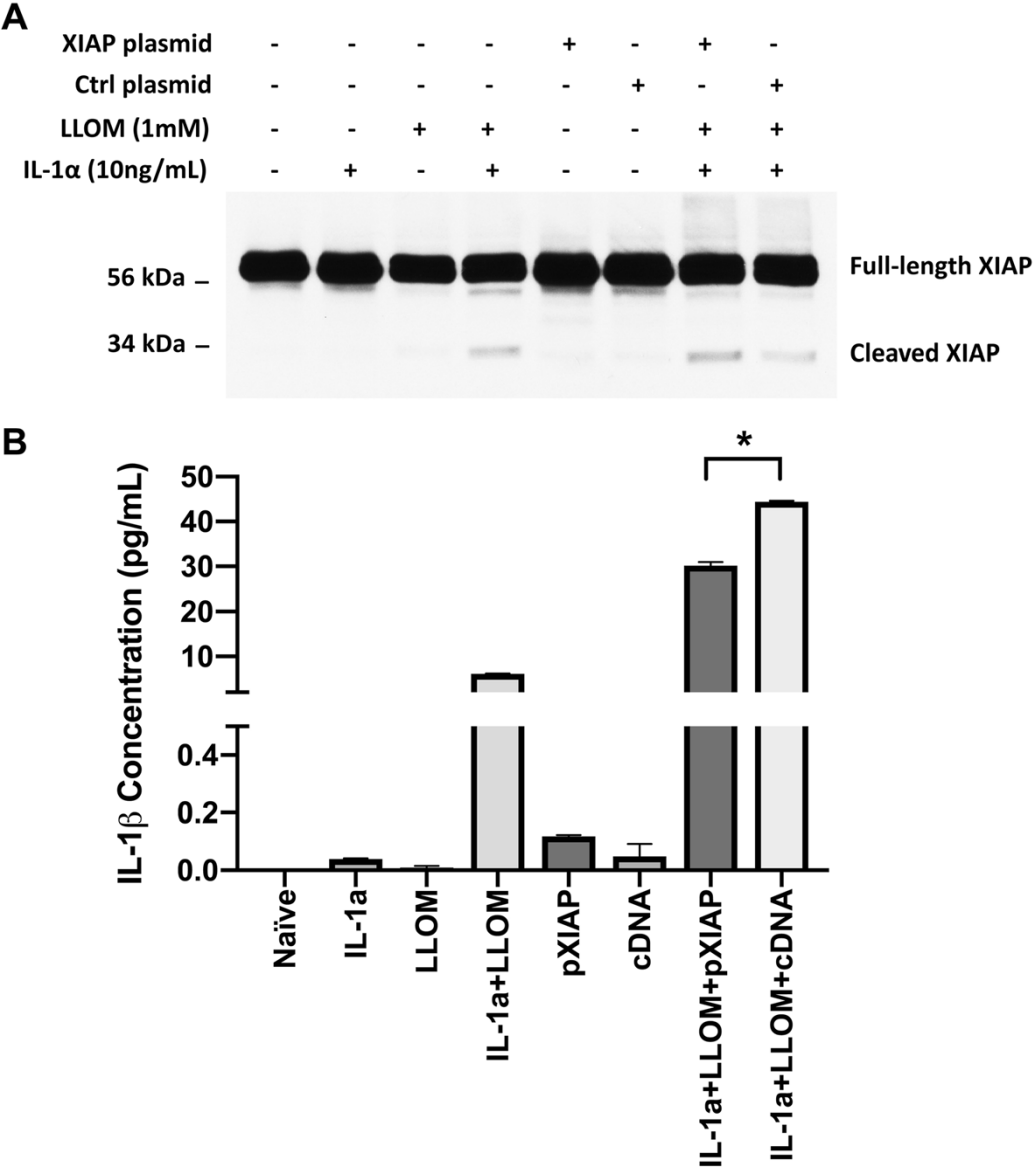
XIAP Overexpression Yields More Cleaved XIAP and Reduces IL-1β Secretion. **a** Transfection of the XIAP plasmid increased the full-length XIAP protein expression in primary RPE cells, which under Leu-Leu-OMe (LLOMe)-induced inflammasome activation, resulted in enhanced XIAP cleavage with lower molecular weight bands at ~ 34 kD. **b** When XIAP full-length protein was cleaved into small bands, significant IL-1β secretion was observed. Primary RPE cells overexpressing the XIAP full-length protein were found to have a significantly lower level of IL-1β secretion under inflammasome activation, compared to cells transfected with vector control (One-way ANOVA, *p < 0.05)

Next, we studied the kinetics between the decline of XIAP protein and the secretion of IL-18 and IL-1β. RT-PCR assays were used to select the lowest effective dose of XIAP siRNA after a 48-h transfection period. It appeared that XIAP siRNA was effective at 2.5 nM, the lowest of all three concentrations tested, causing a reduction in XIAP mRNA level of more than 75% (Fig. 6a and Additional file 2: Figure S2) and a dramatic decrease in XIAP protein level (Fig. 6b). When combined with Leu-Leu-OMe stimulation, the 2.5-nM XIAP siRNA induced an even greater depletion of XIAP protein in ARPE-19 cells (Fig. 6b), compared to non-stimulated cells. Concomitantly, we saw a steady increase of released IL-18 in the culture supernatants of XIAP siRNA silenced, Leu-Leu-OMe stimulated ARPE-19 cells, compared to those with only Leu-Leu-OMe stimulation (Fig. 6b). The Leu-Leu-OMe induced XIAP cleavage was ameliorated by XIAP siRNA (Fig. 6c) but enhanced by XIAP overexpression (Fig. 7a), indicating the 34 kDa bands were specific fragments of the full-length XIAP protein. Similar to the trend of IL-18 secretion in ARPE-19 cells, XIAP siRNA incubation greatly enhanced IL-1β secretion in primary RPE cells when it was combined with IL-1α and Leu-Leu-OMe stimulation, compared to either the IL-1α + Leu-Leu-OMe group or the Ng siRNA + IL-1α + Leu-Leu-OMe group (Fig. 6d). However, in the presence of sufficient XIAP protein, IL-1β secretion was downregulated in primary RPE cells, compared to those transfected with the vector control (Fig. 7b). Taken together, these data indicate that inflammasome-mediated IL-18 and IL-1β release is regulated by XIAP levels.

## Discussion

### XIAP: more than just an anti-apoptotic factor

**Fig. 8:**
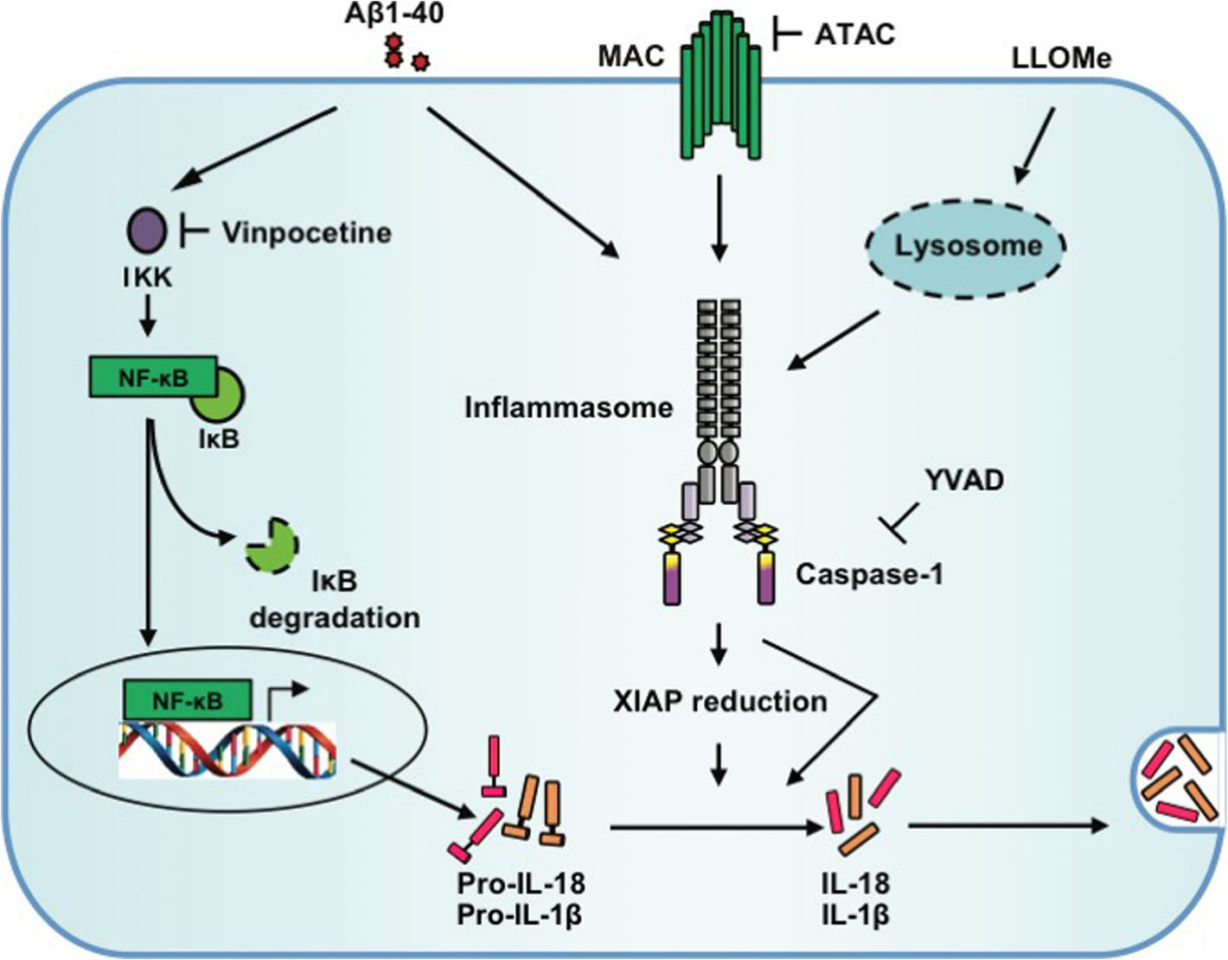
Schematic diagram of proposed interactions between XIAP and the inflammasome cascade. In our previous animal studies, the inflammasome can be activated by either amyloid beta (Aβ) or membrane attack complex (MAC). Inhibition of either the NF-κB pathway (by vinpocetine) or the MAC deposition (by ATAC) is sufficient to prevent inflammasome activation. Reduced XIAP protein levels are found both in vitro (by LLOMe-mediated lysosomal destabilization) and in vivo (as shown in Fig. 1) upon inflammasome activation and can be reversed by ATAC (the “activating” step), not vinpocetine (the “priming” step). Inhibition of Caspase-1 cleavage by YVAD also reduced the cleavage of XIAP and IL-1β secretion. When RPE cells have reduced XIAP (e.g., silenced by siRNA interference) prior to the activation of inflammasome, more IL-18 and IL-1β will be secreted. However, if XIAP is adequate (e.g., overexpressed by plasmid transfection), a lower level of IL-1β secretion is observed

XIAP is well known for its role in regulating apoptosis. In fact, it is even considered as the most potent caspase inhibitor in vitro [22]. However, compared to its anti-apoptotic function, little is known about XIAP’s involvement in immune regulation. Mutations in the XIAP gene were found to result in a primary immunodeficiency condition in humans, named X-linked lymphoproliferative syndrome 2 (XLP2, OMIM entry number: 300635) [23], while XIAP polymorphisms are responsible for idiopathic periodic fever [24], suggesting that XIAP may play a key role in immune homeostasis. In a recent review, Beug et al. further examined the relationship between the members of inhibitors of apoptosis proteins family (IAPs) and inflammasome activity [25]. Beug et al. conclude that IAPs are involved in many aspects of the innate and adaptive immunity, either through the regulation of NF-κB and MAPK pathways or through the control of inflammasome activity. So far, examples of IAPs, in particular XIAP, regulating inflammasome activity have been reported mainly in immune cells [13, 15, 26]. However, research shows that inflammasome activation is a fundamental defense mechanism used by not only immune cells but also epithelial cells [1, 27, 28]. Indeed, several research groups have independently demonstrated the existence of inflammasome activity in RPE, a type of ocular epithelial cells [4, 6, 29–35]. But none of those studies have looked into the question of whether XIAP regulates the assembly or activity of the inflammasome. In this study, we sought to investigate an inverse relationship between XIAP and inflammasome activation from one of our established animal models of AMD [5]. By comparing our data with two other models [16, 17], we were able to map XIAP into the inflammasome’s activation signal pathway (Figs. 1 and 8) and to exclude the involvement of the NF-κB priming pathway in XIAP-inflammasome interactions.

### Inflammasome activity in RPE vs immune cells

We found that experimental conditions required in this study also affected the activation of the inflammasome, particularly as measured by IL-1β secretion. Primary RPE cell were generally more robust in their ability to secrete IL-1β after inflammasome activation than the cell line ARPE-19. We also observed that incubation in serum-free medium, for 1 h (necessary for caspase-1 inhibition, Fig. 5) or 48 h (necessary for XIAP siRNA, Fig. 6d), often resulted in a lowered secreted level of IL-1β. As fetal bovine serum-free culture medium reduces the proliferation and survival of cultured cells, it is possible that RPE cells kept in serum-free medium for 48 h may have lower abilities to produce/secrete cytokines, which could account for the lowered levels seen in Fig. 6 d compared to Figs. 5 and 7 b [36].

It is also important to point out differences we observed between the RPE, a non-immune ocular cell type compared to immune cells, such as macrophages, dendritic, bone marrow-derived cells that generally secrete orders of magnitude greater levels of IL-1β [6, 26, 27, 37–39]. Differences in conditions that promote activation of the inflammasome in RPE vs immune cells are evident from our work. For example, in Additional file 3: Figure S3, the XIAP siRNA treatment on the primed RAW264.7 cells leads to caspase-1 cleavage with or without ATP activation, when the same LPS/ATP treatment was not adequate to induce caspase-1 cleavage. This was not the case in RPE cells, where LPS/ATP combined stimulation would not trigger caspase-1 cleavage with or without XIAP siRNA (unpublished data). Therefore, intrinsic differences between immune cells and RPE likely underlie the criteria required for inflammasome “priming” in RPE. Our future studies will probe this in more detail.

### Inflammasome-mediated XIAP reduction promotes IL-18 and IL-1β secretion

To address the question of whether XIAP functions as a positive or negative regulator, we performed XIAP siRNA knockdown experiments on RPE cells under inflammasome activation. We showed that knockdown of XIAP strengthens the cells’ capacity to secrete IL-18 and IL-1β when inflammasome activation is induced (Fig. 6b, d). Considered counter-intuitive at first, this finding makes sense when we take into account the recent report on human patients diagnosed with XLP2 disease (XIAP deficiency), where strikingly elevated IL-18 concentrations were discovered in these patients’ sera samples which remained high after treatment despite the fact that other pro-inflammatory cytokines returned to the normal range [40]. Moreover, longitudinal examination of these patients revealed marked increase of IL-18 serum concentration, suggesting a clinical association between XIAP deficiency and high IL-18 levels. On the other hand, the heightened level of IL-1β secretion has also been linked with XIAP deficiency in myeloid cells, which is consistent with our findings [41]. Therefore, collectively, these data indicate XIAP is a negative regulator of inflammasome-mediated IL-18 and IL-1β secretion. However, the molecular structural basis that enables the XIAP-mediated regulation of IL-18 and IL-1β secretion remains unknown and warrants future investigation.

In addition to the altered cytokine secretion, we also reported an inverse correlation between caspase-1 cleavage and XIAP protein level. We speculated it was possible that inflammasome activation caused the reduction in XIAP. To support this interpretation, a caspase-1-specific inhibitor, Z-YVAD-FMK, was used to prevent inflammasome activation, which in turn led to significantly diminished XIAP cleavage (Fig. 5). This is consistent with another study where caspase-1 cleavage and XIAP cleavage are both present in injured rat neurons and suppressing caspase-1 cleavage helped to retain XIAP in its full-length form [15]. Moreover, overexpression of XIAP in primary RPE cells followed by inflammasome activation, resulted in more cleaved XIAP but much less secreted IL-1β, suggesting XIAP is influenced by, rather than affects, inflammasome activation itself (Fig. 7). The observed XIAP-caspase-1 relation here is likely not cell type-specific, as we have also shown caspase-1’s cleavage levels were increased by XIAP siRNA knockdown in murine macrophage cell line, RAW264.7 (Additional file 3: Figure S3). Collectively, these findings favor the notion that XIAP is positioned downstream of caspase-1, regulating cytokine secretion in RPE cells (Fig. 8). Whether XIAP is a natural substrate of, or indirectly influenced by, caspase-1 in RPE cells needs further investigation.

## Conclusions

In this study, we identified a novel role of XIAP in regulating IL-18 and IL-1β, both inflammasome-related cytokine secretion in RPE. Using a combined approach of siRNA and protein overexpression, we were able to assess XIAP’s position along the inflammasome activation pathway. The results reported here help provide insights into the biological consequences of inflammasome activation in RPE and reveal the caspase-1/XIAP/IL-18/IL-1β axis as a target for broader applications in AMD biology and treatment design.

## Additional files

Additional file 1: **Figure S1.** Control siRNA transfection does not affect the level of XIAP mRNA. A series of non-targeting control siRNA concentrations as shown was tested. No significant changes in XIAP mRNA level were observed compared to the non-transfected naïve cells. (EPS 80 kb)

Additional file 2: **Figure S2.** XIAP siRNA transfection on primary human fetal RPE cells. At 2.5 nM concentration, the XIAP targeting siRNAs reduced the XIAP mRNA level to less than 50% of untreated controls, as early as 24 h post-transfection. A higher siRNA concentration (5.0 nM) or longer transfection incubation (48 h) did not further reduce XIAP mRNA levels, suggesting the 2.5 nM siRNA concentration and the 24 h incubation period was already effective (* *p*<0.05, one-way ANOVA). (EPS 103 kb)

Additional file 3: **Figure S3.** XIAP siRNA knockdown enhances caspase-1 cleavage in murine macrophage cell line RAW264.7. The murine macrophage RAW264.7 cell line was used as a positive control cell type to study the relationship between caspase-1 cleavage and XIAP protein level. Under the same LPS/ATP combined stimulation, RAW264.7 cells pretreated with 10 nM XIAP siRNA showed enhanced cleavage of full-length pro-caspase-1 (45 kD) into cleaved caspase-1 bands (20 kD). (EPS 2163 kb)

## Data Availability

All data generated or analyzed during this study are included in this published article.

## Abbreviations

AIM2: Absent in melanoma 2
AMD: Age-related macular degeneration
ATAC: Aurin tricarboxylic acid complex
DAMPs: Danger-associated molecular patterns
GAPDH: Glyceraldehyde 3-phosphate dehydrogenase
GSDMD: Gasdermin D
IAPs: Inhibitors of apoptosis proteins family
IL-18/1α/1β: Interleukin-18/1α/1β
Leu-Leu-OMe: L-leucyl-L-leucine methyl ester
MAC: Membrane attack complex
NLRC4: NLR family CARD domain containing 4
NLRP1: NLR family pyrin domain containing 1
NLRP3: Nod-like receptor protein 3
RPE: Retinal pigment epithelium
RT-PCR: Reverse transcription polymerase chain reaction
XIAP: X-linked inhibitor of apoptosis protein
XLP2: X-linked lymphoproliferative syndrome 2
